# Combination of Calcitriol and Zoledronic Acid on PINP and *β*-CTX in Postoperative Patients with Diabetic Osteoporosis: A Randomized Controlled Trial

**DOI:** 10.1155/2022/6053410

**Published:** 2022-06-03

**Authors:** Qingchang Hu, Qi Wang, Fan Liu, Lishuai Yao, Lei Zhang, Guangdong Chen

**Affiliations:** ^1^Department of Emergency Medicine, Cangzhou Central Hospital, Cangzhou, China; ^2^People's Hospital of Qingxian, Qingxian, China; ^3^Anesthesia Operating Room, Cangzhou Central Hospital, Cangzhou, China; ^4^Department of Orthopaedics, Cangzhou Central Hospital, Cangzhou, China

## Abstract

**Objective:**

To explore the effect of calcitriol combined with zoledronic acid in posterior cruciate ligament tibial avulsion fractures of the knee joint in patients with diabetic osteoporosis.

**Methods:**

Between January 2020 and January 2022, 60 patients with diabetic osteoporosis treated in our hospital were included. All patients underwent knee joint posterior cruciate ligament tibial avulsion fractures, and they were randomized (1 : 1) into the observation group (calcitriol combined with zoledronic acid) and control group (calcitriol). The two groups were compared with respect to the improvement of bone mineral density and bone metabolism indexes, the pain degree (VAS) and knee joint function (Lysholm), and the incidence of refracture.

**Results:**

Both groups showed an increasing bone mineral density after treatment, and significant increase was observed in the observation group vs. control group (all *p* < 0.05). After treatment, VAS scores decreased in the two groups, and Lysholm scores increased compared to the corresponding values before treatment (all *p* < 0.05), with more notable changes in the observation group versus control group (all *p* < 0.05). The observation group had fewer cases of refractures than the control group (2 cases vs. 8 cases) (*p* < 0.05).

**Conclusion:**

Calcitriol combined with zoledronic acid used in patients with diabetic osteoporosis after the posterior cruciate ligament tibial attachment avulsion fracture of the knee joint yields a promising result in enhancing bone mineral density and bone metabolism indicators, relieving pain, improving knee joint function, and reducing the risk of refracture.

## 1. Introduction

Diabetes is a chronic metabolic disease with high incidence rate and disability rate. Long-term hyperglycemia stimulation may cause disorder of calcium and phosphorus metabolism in bone tissue, resulting in bone remodeling and bone microstructure damage and further undermining bone quality. The bulk of evidences recognize diabetic disease as an independent risk factor for osteoporosis. Diabetic osteoporosis, a serious complication of diabetes, is a systemic bone metabolism disease characterized by hyperglycemia, hyperinsulinemia, osteopenia, reduced bone turnover rate, and increased bone fragility [1–3]. Diabetic patients with osteoporosis are more prone to fracture in lumbar spine due to the cancellous bones, with the major presentations of varying degrees of activity limitation and severe pain. They are susceptible to knee joint dysfunction in case of external force, of which, the avulsed tibial end of the cruciate ligament is frequently seen. Clinically, the mainstay is arthroscopic surgery, which helps maintain stable knee joint function and improve the quality of life of patients [4]. However, there is a higher risk of refracture after surgery due to the patient's own osteoporosis together with the reduction of bone mass and activity. In this regard, standardized adjuvant treatment after surgery should be urgently addressed. Calcitriol is a basic drug for the treatment of osteoporosis, which is beneficial to the absorption of calcium in the intestine and intake of calcium [5]. Zoledronic acid, a special type of bisphosphonate, inhibits the function of osteoclasts, reduces bone resorption, and enhances the quality of vertebral bone [6]. Nevertheless, the combination of the two has yet been investigated. Accordingly, this study was designed to explore the combination of calcitriol and zoledronic acid in osteoporosis surgery by analyzing 60 cases of diabetic osteoporosis patients with knee joint posterior cruciate ligament tibial avulsion fractures.

## 2. Study Design and Participants

### 2.1. Participants

Totally 60 cases of diabetic osteoporosis patients undergoing surgery for avulsion fractures of the posterior cruciate ligament of the knee in our hospital from January 2020 to January 2022 were selected and randomized at a ration 1 : 1 via random envelopes into the observation group (male : female = 16 : 14; aged 43-78 years old (62.59 ± 7.32) years; body mass index (54.32 ± 5.03) kg (44~75 kg); glycosylated hemoglobin: (6.49 ± 2.22) %, (6.51 ± 2.10) %; the cause of fracture: 15 cases of sports injury, 7 cases of traffic accidents, 6 cases of accidental falls, and 2 cases of others) and the control group (male : female = 17 : 13; aged 40-77 (62.31 ± 7.57) years old; body mass index (54.28 ± 5.10) kg (43~78 kg); the cause of fracture: 14 cases of sports injuries, 7 cases of traffic accidents, 7 cases of accidental falls, and 2 cases of others). The baseline information were well balanced in the two groups (*p* > 0.05).

### 2.2. Inclusion and Exclusion Criteria

Inclusion criteria were as follows: (1) the patients were diagnosed with osteoporosis by X-ray diagnosis and imaging examination [5], and all underwent tibial avulsion fractures of the posterior cruciate ligament of the knee joint; (2) met the diagnostic criteria of WHO diabetes in 1999, (3) patients and their family members were informed of the purpose and significance of the study before the study, signed the consent form on the premise of understanding the purpose of the study, and the medical ethics committee authorized the study before the commencement of the study, (4) with normal communication ability, and (5) with complete clinical data. Exclusion criteria were as follows: (1) with damaged vital organs; (2) with mental disorders or unconsciousness; (3) cancer patients; (4) with other types of fractures; (5) poor coordination or unable to follow the rules of the study; (6) allergies or intolerance to the study drugs; (7) with immune system diseases or abnormal blood tests; and (8) with thyroid disease, osteoarthritis, or rheumatoid arthritis, which can cause osteoporosis.

### 2.3. Methods

All patients were treated with routine control of blood glucose. Fasting blood glucose was measured once a week to ensure good blood glucose control. The control group was as follows: patients were treated with calcitriol capsules (CHIN TENG Pharmaceutical Industrial Co., Ltd., approval number HC20171016, specification: 0.25 *μ*g × 10 s) after operation, at a dose of 0.25 *μ*g each time, 1 time/d. The observation group was as follows: on the basis of the control group, zoledronic acid injection (Novartis Pharma Stein AG, approval number H20123153, specification: 100 mL: 5 mg (Yigu)) dissolved with 100 mL normal saline was administered via intravenous infusion, at a speed of 15 minutes or more, and the treatment was performed once in a year.

### 2.4. Observation Index

After a year of treatment, the improvement of the patient's bone mineral density and bone metabolism index before and after treatment was detected; the pain degree and the improvement of knee joint function were evaluated; whether the patient has refracture after operation was observed. (1) The bone density test mainly includes the greater trochanter and the neck of the femur, using the South Korean Auster dual-energy X-ray bone densitometer EXA-3000 (Shanghai Sanwei Medical Equipment Co., Ltd.). (2) 3 mL fasting in the morning peripheral venous blood was collected and centrifuged at 3000 r/min, and then the samples were processed for a total of 10 minutes to isolate the serum. Serum PINP indicators and *β*-CTX were detected by chemiluminescence immunoassay, and the kit was purchased from Shanghai Qiming Biotechnology Co., Ltd. (3) The VAS scale was used to evaluate the degree of pain in patients. The scale was marked on a 10 cm moving scale, and the left and right ends represent, respectively, no pain (0 point) and severe pain (10 points), mild pain (1 to 3 points): slight pain but does not affect sleep and life and can be tolerated; moderate pain (4 to 6 points): obvious pain and need to use analgesics to help sleep; and severe pain (7 to 10 points): severe pain and intolerable, seriously affects sleep, and even results in passive posture and neurological disorders [6]. (4) Lysholm scale was used to evaluate knee joint function, including claudication, support, instability, and swelling and other 8 items. The total score is 100 points. A score of <70 indicates that the knee joint function is affected. A higher score indicates better knee joint functions [7]. (5) One year after treatment, all patients underwent X-ray examination to assess the rate of re-fracture. All the above indicators were collected by researcher blind to the grouping.

### 2.5. Statistical Analysis

Statistical analysis was done by using SPSS22.0 software package. The counting data and measurement data were expressed as (%) and (*x* ± *s*), respectively, and processed using *X*^2^ and *t*-test, respectively. A *p* value of <0.05 indicates that the difference is statistically significant.

## 3. Results

### 3.1. Comparison of the Bone Mineral Density

Both groups showed an increased bone mineral density after treatment, and the increase value in the observation group was more notable (*p* < 0.05). Before treatment, no distinctive difference was observed in bone mineral density between the two groups (*p* > 0.05), see Table 1.

### 3.2. Comparison of Bone Metabolism Indexes

The *β*-CTX and PINP indexes of patients in the two groups were not statistically different before treatment (*p* > 0.05). After treatment, the indexes in two groups were lower than the corresponding values before treatment, with significant reduction in the observation group (*p* < 0.05), as shown in Table 2.

### 3.3. Comparison of VAS Scores and Lysholm Scores

After treatment, significant decrease was observed in VAS scores in both groups, whereas significant increase was observed in Lysholm scores when compared to those before treatment (all *p* < 0.05), and the changes was more greater in the observation group versus control group (all *p* < 0.05), see Table 3.

### 3.4. Comparison of the Incidence of Refracture

The observation group had fewer number of refractures than the control group (2 cases vs. 8 cases) (*p* < 0.05, Table 4).

## 4. Discussion

As people ages, the metabolic function decreases; also, less amount of exercise and changes in dietary structure results in bone density decline. All these easily lead to diabetic osteoporosis. Diabetic osteoporosis, with insidious clinical manifestations in the early stage, can be painful and easy to fracture as the disease progresses. Among them, posterior cruciate ligament tibial attachment avulsion fracture is a very common type, which not only negatively affects knee joint function but also accelerates the degeneration of the knee joint [8]. To our best understanding, early surgery benefits fracture reduction and prognosis of patients. In recent years, studies have found that patients with posterior cruciate ligament tibial attachment avulsion fractures accounts for 70% to 80%, which is presumably related to postoperative fractures and loosening of the prosthesis. Knowingly, osteoporosis patients suffer reduced bone mass after surgery and stress changes. Therefore, despite the improved motor function, the diabetic osteoporosis has not been fundamentally resolved. Therefore, postoperative treatments need to be supplemented to improve patient prognosis and prevent refracture.

Normally, diabetic osteoporosis patients suffer pain after surgery and require immobilization, during which bone loss is likely to occur, and thus bone density enhancement should be accordingly emphasized to accelerate the recovery of patients' mobility. Calcitriol, a metabolite, is a commonly used drug for the treatment of osteoporosis and mainly metabolized by the liver and kidneys. Its product 1-25 dihydroxyvitamin D3 can promote the absorption of calcium and regulate the balance of Ca2+, which is conducive to fracture synthesis, promotes the increase of bone density and bone mass by stimulating the activity of osteoblasts, and plays a role in alleviating osteoporosis [9]. However, the single use of calcitriol yields limited effectiveness, and adverse reactions such as hypercalcemia and malnutrition are prone to occur. In this study, the observation group was treated with zoledronic acid plus alcitriol, and the results were promising. As a bisphosphonate drug, after being absorbed by osteoclasts, zoledronic acid inhibits the activity and synthesis of osteoclasts, accelerates their apoptosis, reduces the number of osteoclasts, and significantly improves bone resorption. Additionally, after the drug is combined with hydroxyapatite crystals, it can effectively block the adsorption of osteoclasts to the bone surface, inhibit its biological activity on the bone surface, reduce bone resorption, and promote the improvement of bone density [10]. Remarkably, the results of this study found that the two groups of patients had increased greater trochanter and femoral neck bone density after treatment, suggesting that both methods are effective in improving bone density. Interestingly, the increase in the observation group is greater, indicating that the combination of zoledronic acid generates more excellent results.

Previous study pointed out that most patients with diabetic osteoporosis have abnormal bone metabolism. As important markers of bone formation in the human body, *β*-CTX and PINP are highly expressed in patients with osteoporosis, and it has been used in the evaluation of osteoporosis [11‒13]. In this study, the observation group outperformed the control group in the improvement of bone metabolism indicators, confirming the effect of zoledronic acid in improving the level of bone metabolism. Zoledronic acid can directly acts on the surface of bones, ensuing hydroxyphosphoric lime crystals to deposit on the bone, which is conducive to the formation of new bone, healing of fractures, and pain relief and prevention of refractures. According to our study results, superior performance was observed with respect to VAS score and Lysholm score in the observation group, with lower incidence of refracture, indicating a good safety profile. However, the present study conclusions might be moderated to null due to the limited experimental duration and smaller sample size. Therefore, further studies with larger sample size and longer follow-up are required to verify and replicate our results.

Taken together, the combination of calcitriol and zoledronic acid is an alternative for patients with diabetic osteoporosis knee joint posterior cruciate ligament tibial anchor avulsion fractures, with respect to the improvement of bone density and bone metabolism, pain relief, and knee joint function enhancement and reduction of refracture risk.

## Figures and Tables

**Table 1:** Comparison of bone mineral density between the two groups ($\overset{—}{x}\pm s$, mg/cm^3^).

Groups	*n*	Greater trochanter		Femoral neck	
		Before treatment	After treatment	Before treatment	After treatment
Observation group	30	0.63 ± 0.02	0.86 ± 0.13^∗^	0.65 ± 0.06	0.89 ± 0.14^∗^
Control group	30	0.62 ± 0.03	0.75 ± 0.14^∗^	0.63 ± 0.05	0.73 ± 0.16^∗^
*t*	/	1.519	3.154	1.403	4.122
*p*	/	0.134	0.003	0.166	<0.001

Note: compared with the same group before treatment, ^∗^*p* < 0.05.

**Table 2:** Comparison of bone metabolism indexes between the two groups ($\overset{—}{x}\pm s$).

Groups	*n*	*β*-CTX (ng/mL)		PINP (ng/mL)	
		Before treatment	After treatment	Before treatment	After treatment
Observation group	30	0.47 ± 0.03	0.20 ± 0.02^∗^	38.26 ± 3.04	15.05 ± 2.15^∗^
Control group	30	0.45 ± 0.09	0.27 ± 0.04^∗^	38.37 ± 3.18	19.54 ± 2.36^∗^
*t*	/	1.155	8.573	0.137	7.703
*p*	/	0.253	<0.001	0.892	<0.001

Note: compared with the same group before treatment, ^∗^*p* < 0.05.

**Table 3:** Comparison of VAS scores and Lysholm scores between the two groups ($\overset{—}{x}\pm s$, point).

Groups	*n*	VAS		Lysholm	
		Before treatment	After treatment	Before treatment	After treatment
Observation group	30	6.32 ± 1.04	1.84 ± 0.14^∗^	48.94 ± 4.43	78.42 ± 6.57^∗^
Control group	30	6.29 ± 1.01	2.96 ± 0.23^∗^	49.32 ± 4.52	68.34 ± 5.77^∗^
*t*	/	0.113	22.783	0.329	6.314
*p*	/	0.910	<0.001	0.743	<0.001

Note: compared with the same group before treatment, ^∗^*p* < 0.05.

**Table 4:** Comparison of the incidence of refracture between the two groups (%).

Groups	*n*	Number of refractures	Ratio
Observation group	30	2	6.7
Control group	30	8	26.7
*X* ^2^	/	4.320	
*p*	/	0.038	

## Data Availability

The datasets used during the present study are available from the corresponding author upon reasonable request.
